# Synthetic biology advances for pharmaceutical production

**DOI:** 10.1016/j.copbio.2015.02.004

**Published:** 2015-12

**Authors:** Rainer Breitling, Eriko Takano

**Affiliations:** Manchester Centre for Synthetic Biology of Fine and Speciality Chemicals (SYNBIOCHEM), Manchester Institute of Biotechnology, Faculty of Life Sciences, University of Manchester, 131 Princess Street, Manchester M1 7DN, United Kingdom

## Abstract

Synthetic biology enables a new generation of microbial engineering for the biotechnological production of pharmaceuticals and other high-value chemicals. This review presents an overview of recent advances in the field, describing new computational and experimental tools for the discovery, optimization and production of bioactive molecules, and outlining progress towards the application of these tools to pharmaceutical production systems.

## Highlights

• Synthetic biology is quickly moving from proof of concept to industrial application.
• Pharmaceuticals are a promising target for advanced genetic engineering.
• Genome sequence data indicate vast underexploited biosynthetic capacity.
• Synthetic biology can create libraries of novel chemicals enriched for bioactivity.
• Synthetic biology expands the range of available chassis organisms for industry.

## Graphical abstract

**Figure:**
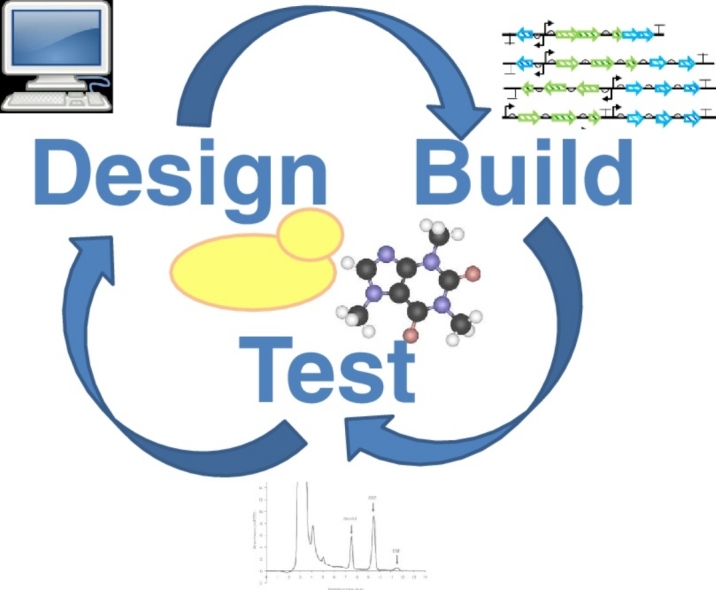

**Current Opinion in Biotechnology** 2015, **35**:46–51

This review comes from a themed issue on **Pharmaceutical biotechnology**

Edited by **Guillermo de la Cueva-Méndez** and **Dror Seliktar**

For a complete overview see the Issue and the Editorial

Available online 3rd March 2015

**http://dx.doi.org/10.1016/j.copbio.2015.02.004**

0958-1669/© 2015 The Authors. Published by Elsevier Ltd. This is an open access article under the CC BY license (http://creativecommons.org/licenses/by/4.0/).

## The synthetic biology revolution

Synthetic biology has seen rapid advances in the last couple of years. Initially focusing on proof-of-concept studies illustrating our ability of writing genetic code on a large scale and demonstrating the usefulness of introducing engineering concepts into biology, the field is now quickly moving towards industrial applications [1–7]. In particular, the engineering of microbial production systems for high-value small molecules is seen to hold great potential, aiming at compounds that range from flavours and fragrances to clinically relevant pharmaceuticals [8]. While just a few years ago, synthetic biology was seen as an avant-garde concept, its ideas and methods have now largely entered the mainstream of molecular biology and genetic engineering, so much so that the necessity for its ambitious engineering metaphors is already being debated [9,10]. In this review we provide a concise overview of some of the early achievements in the synthetic biology of pharmaceuticals and the advances in tools (molecular and computational) that are expected to drive this field forward rapidly.

## Synthetic biology for pharmaceuticals

Pharmaceuticals have inspired some of the earliest success stories of synthetic biology for two main reasons: on the one hand, small-molecule drugs in current use (from aspirin to artemisinin) are very often derived from natural products, so that a return to microbial production systems is seen as relatively straight-forward. On the other hand, many natural biosynthetic pathways show a surprising level of built-in modularity at many levels, which can be exploited by the engineering approaches of synthetic biology [11,12]. This is particularly true for the large group of bioactive natural products that are active as antibiotics and related compounds: the evolutionary requirements for rapid diversification and for robust cross-species compatibility of pathways that are constantly exchanged between host organisms have shaped large modular assembly lines that can serve as starting points for synthetic biology. Cummings *et al*. [13] and Poust *et al*. [14] have recently reviewed the possibilities and limitations of this approach with a particular focus on polyketide synthases, and Kittleson *et al*. examine the challenges of modular genetic engineering from a broader systems biological perspective [15].

The synthetic biology of pharmaceuticals is further inspired by the recent avalanche of microbial genome and metagenome sequences which revealed an unexpected richness of unexplored biosynthetic capacities in almost every genome analysed [16,17]. For example, the recently published first comprehensive assessment of secondary metabolite diversity across microbial kingdoms, based on the computational analysis of more than 1100 complete genome sequences, detected more than 30 000 putative biosynthetic gene clusters (estimating a false-discovery rate of 5%) [18]. These were broadly distributed across the phylogenetic tree, with clusters of particular richness in, for example, actinomycetes, *Burkholderia* and *Pseudomonas*, but ‘talented’ strains of high predicted biosynthetic capacity being found in almost every larger bacterial group. A targeted analysis of Actinobacteria, combining genome mining and metabolomics, concluded that this group alone encodes for hundreds of thousands of possible drug leads [19^••^]. Under standard conditions, the majority of this biosynthetic potential is silent or cryptic, and synthetic biology is seen as a potential tool for awakening and mining this rich source of potential drug candidates on a large scale [17,20–24]. Additional genetic engineering can then be applied to obtain variations and further diversity, with new or improved bioactivity [25,26].

## Tools for the synthetic biology of pharmaceuticals

Synthetic biology, like all engineering disciplines, relies on the availability of powerful standardized tools for all steps along the design–build–test (and learn) cycle. Many of the required tools, for example genome synthesis, assembly and editing methods [27–29], are broadly generic. Others are specific to the field of small-molecule synthetic biology. These begin with methods for the comprehensive discovery and annotation of biosynthetic building blocks in newly sequenced genomes [30], as exemplified by the antiSMASH pipeline [31^•^,32]. In combination with advances in mass spectrometry-based analytics these annotations can be used to link genome information directly to bioactive compounds observed in bacterial cultures: this has recently been demonstrated for peptidic metabolites (both non-ribosomal peptides and Ribosomally synthesized and posttranslationally modified peptides, RIPPs), where incomplete and noisy sequence information derived from mass spectrometry can be used to successfully identify the gene clusters likely to produce them, and even to determine the actual structure of the end product [33,34^••^].

Another exciting source of biosynthetic building blocks is promised by recent advances in the de novo computational design of enzymes with activities that were previously unavailable in biologicals systems [35]. Enzyme engineering approaches also benefit from the increasing availability of genome sequences, which allow evolution-guided manipulation of existing enzymes, for example, for altered substrate specificity in molecular assembly lines for polyketides or non-ribosomal peptides [36^•^,37]. For example, identification of the recombination events underlying a major switch in substrate specificity during evolution of a bacterial non-ribosomal peptide synthase, allowed Crüsemann *et al*. [36^•^] to create enzyme variants that accepted alternative substrates, based on the inferred recombination points.

Analogous evolutionary strategies can also be applied at the pathway level, as demonstrated by the recent rational design of novel functional polyketide synthases by emulating the natural evolutionary processes underlying the diversification of this biosynthetic class [38^•^]. By examining the shared evolutionary history of the polyketides aureothin, spectinabilin and luteoreticulin, it was possible to develop a rational strategy of recombination and domain exchanges that reprogramme the aureothin polyketide biosynthetic pathway into a pathway that produced luteoreticulin. Protein engineering can also be very useful at the small scale, for instance creating enzymatic building blocks that are not affected by the end-product inhibition that is usually seen in natural enzymes. Schendzielorz *et al*. recently demonstrated the power of this ‘mutein’ approach in a case study on amino acid biosynthesis in *Corynebacterium glutamicum*, where high-throughput removal of inhibition allowed a massive increase in production titres [39^••^].

Other approaches to diversifying the pool of available chemical building blocks rely on a variety of experimental approaches. Walker *et al*. used synthetic biology to develop a library of new fluorinated building blocks for biomolecules [40^••^]. Starting from the fluoroacetate pathway of *Streptomyces cattleya*, the only known natural source of fluorinated biomolecules, they engineered acetate-based polyketide biosynthesis to incorporate the fluorinated precursor, via synthesis of fluoromalonyl-CoA as a modified extender unit. As many other natural products, ranging from isoprenoids to steroids and alkaloids, are also acetate-derived, this strategy offers potentially a general approach for expanding the chemical space around known pharmaceuticals.

The production of small molecules, whether awakened from genome information or optimized in a native or heterologous host, requires not only expression of the core biosynthetic pathway, but also sufficient supply of precursors and reduced competition from alternative reactions. Computational modelling has been useful for this purpose, and the specific challenges of modelling for secondary metabolite production have recently been reviewed [41]. The increasing availability of automated model construction and curation tools further increases the accessibility of the technology [42] and enables, for example, the comprehensive computational survey of potential production hosts for heterologous pathways [43].

Other computational tools allow the design of the actual pharmaceutical production system. A particularly ambitious recent example is the Retropath tool for the principled design of entire metabolic circuits [44^••^], based on constraining information about the metabolic capacities of the envisaged host organism (chassis) and the scope of available chemical reactions. In addition to the production modules, the Retropath framework also allows exploration of the design space for biosensing and regulation of the synthetic pathways, with the ultimate aim of enabling the construction of smart therapeutics, which integrate pharmaceutical synthesis and point-of-need delivery. Proof-of-concept examples of similar devices have recently been introduced, including engineered *Escherichia coli* that can sense and kill *Pseudomonas aeruginosa* biofilms by eavesdropping on the target quorum sensing signals and releasing antibiotic pyocin proteins in response [45].

While much of the actual DNA-level building of engineered systems for pharmaceutical production relies on generic tools, it is especially reliant on emerging methods for the large-scale assembly of libraries of biosynthetic pathways, to explore chemical space around existing bioactive compounds or to discover new activities based on hybrids and module-shuffled assemblies of biosynthetic building blocks from various sources. A focused review of DNA assembly methods available for this particular challenge has recently been provided by Cobb *et al*. [46^•^]. Recent developments in the use of phage integrases for the multiplexed pathway assembly offer another important contribution to the toolbox of synthetic biology especially for the engineering of biosynthetic libraries [47^••^].

Moreover, the development of universal transfer and expression systems specifically for large biosynthetic gene clusters shuttled between species facilitates the exploration of bioactive molecules from a variety of sources. For example, Loeschke *et al*. introduced a systems that combines conjugation-based DNA transfer, randomized transposition-based integration into the host genome, and T7 RNA polymerase-driven bidirectional transcription for concerted gene expression [48]. Applications to pigmented secondary metabolites, zeaxanthin and prodigiosin, demonstrated the utility of this system in different bacterial species. A similar system, specifically for bioprospecting in filamentous fungi, was recently presented by Unkles *et al*. [49].

The design–build–test cycle is closed by the debugging of the engineered microbes, for example, by high-resolution metabolomics, which allows not only the quantitation of product levels, but more importantly a global assessment of metabolic bottlenecks and potentially deleterious side reactions [50–52]. The insights gained at this step are then available to drive another iteration of the cycle, improving the predictive models and informing the next steps in pathway engineering.

## Applications of synthetic biology for pharmaceuticals

The first and most widely publicized application of synthetic biology for the industrial production of an important drug is the case of semi-synthetic artemisinin. This antimalarial drug was originally obtained from plant sources but can now be produced at large quantities in heterologous hosts, including *Saccharomyces cerevisiae* [53]. The project leading to the commercially viable engineered strains took almost ten years and established a large number of basic concepts and technologies required for the successful design of biosynthetic systems for pharmaceutical compounds [54], ranging from optimization of the chassis organism for precursor supply (in this case, artemisinic acid, which proved to be unavailable in sufficient amounts even in engineered strains of the originally intended production host *E. coli*), to the importance of eliminating or reducing unwanted side reactions (such as the production of reactive oxygen species, which severely limited the viability of early artemisinic acid producing yeast strains). The ability to pursue many design routes in parallel allowed the exploration of additional useful concepts, for instance the use of engineered protein scaffolds to optimize the relative activities of enzymes along a designer production pathway [55], or the importance of subtle balancing of enzyme activities along a pathway for optimal production [56,57^••^].

Synthetic biology for other pharmaceuticals can now build on the experiences of the artemisinin project, and consequently the last few years have seen rapid developments in the field, not only for closely related isoprenoids, such as taxol [58], farnesene [59] and many others [58,60–62].

Jaitzig *et al*. demonstrated the possibility of heterologous expression of the huge molecular assembly lines required for pharmaceutically interesting natural products in the most attractive production host, *E. coli* [63]. They expressed the entire 654 kDa non-ribosomal peptide synthase for valinomycin in soluble and active form, creating a valuable system to produce analogues of the drug by making full use of the genome engineering methods available for *E. coli*.

Shao *et al*. applied the refactoring strategy of synthetic biology for awakening a silent biosynthetic pathway [64]: by removing all native regulatory control of the spectabilin cluster of *Streptomyces orinoci* and replacing it with a system of constitutive and inducible heterologous promoters in a plug-and-play scaffold, they achieved the straight-forward production of the end product at detectable levels amenable for analysis and further optimization.

Klein *et al*. harnessed the power of synthetic biology in yeast, *S. cerevisiae*, to create an entire library of 74 novel chemical compounds enriched for bioactive compounds [65^••^]. Using randomly assembled biosynthetic pathways from a variety of natural sources [66] in combination with an internal activity screen in the same cells used for the production, they were able to target the discovery process towards molecules with structural and biophysical properties that comply with traditional rules for drug-likeness.

Important advances have also been made in the establishing of engineered host systems (chassis) that are optimized for the production of pharmaceuticals and other secondary metabolites. Komatsu *et al*. developed genome-minimized variants of the industrial microbe *Streptomyces avermitilis*, which grow faster than the wild type, lacked the principal endogenous biosynthetic gene clusters that often result in precursor competition and analytical complexity, and successfully achieved substantial heterologous expression of a large number of secondary metabolite gene clusters [67]. Nikel *et al*. argue for environmental *Pseudomonas* strains exhibiting favourable metabolic and stress-tolerance properties as promising new hosts for biotechnological applications, based on the increasing ability of synthetic biology to engineer such non-classical model organisms [68]. Given the increasing ease of genome engineering in multicellular organisms, for example, using the CRISPR-Cas system and related technologies [69,70], even plants (and plant cell cultures) are increasingly returning to the focus as potential biotechnological production systems for pharmaceutical compounds, given their versatile endogenous metabolism [71,72].

## Conclusions

The use of microbial production systems for pharmaceuticals and other high-value chemicals is clearly entering a new phase right now. The ambitious engineering aims of synthetic biology are rapidly becoming a reality, based on advances in our ability to edit genomes, identify and optimize biosynthetic building blocks, rapidly create libraries of pathways and novel compounds, and to debug and improve the engineered systems. As has always been the case for natural products research, diversity will remain a key feature: synthetic biology is built on diversity of computational tools, genome engineering methods, analytical and screening technologies, but it also enables new diversity at the level of host organisms and chassis, as well as biosynthetic modules and bioactive end products. All of these come together within the shared framework of the design–build–test cycle of synthetic biology, which in the coming years will move many of the examples indicated in this review from the proof-of-concept stage to wide-spread application in the pharmaceutical industry.

## References and recommended reading

Papers of particular interest, published within the period of review, have been highlighted as:

• of special interest
•• of outstanding interest
